# Vessel Preparation Is Essential to Optimize Endovascular Therapy of Infrainguinal Lesions

**DOI:** 10.3389/fcvm.2020.558129

**Published:** 2020-09-23

**Authors:** François Saucy, Hervé Probst, Rafael Trunfio

**Affiliations:** ^1^Service of Vascular Surgery, Etablissement Hospitalier de la Côte, Morges, Switzerland; ^2^Service of Vascular Surgery, Department Hear and Vessels, University Hospital, Lausanne, Switzerland

**Keywords:** peripheral arterial disease, directional atherectomy, cutting balloon (CB), lumen gain, calcification, scoring balloon catheter

## Abstract

Symptomatic peripheral arterial disease management involves medical treatment and interventional procedures. Intermittent claudication and critical limb threatened ischemia (CLTI) should be individually considered with specific outcomes and procedures. When intervention is required, an endovascular approach is usually the first-line option. Plain balloon angioplasty was previously used to dilate clinically significant femoropopliteal lesions with variable results. However, over recent years, the use of self-expanding nitinol stents has enabled treatment of long lesions, yielding significantly improved clinical results. Drug-eluting technology has also exhibited a capacity to limit in-stent restenosis and to drive target revascularization. Nevertheless, calcifications and elastic recoil of the arterial wall remain risk factors for early restenosis and failure. Therefore, vessel preparation using specific devices is required to modify vessel compliance and debulk obstructive calcification. In this short review, we provide an overview of the options for gaining lumen before stenting or dilation using drug-coated balloons.

## Introduction

Symptomatic atherosclerotic disease of the lower limbs may occur as intermittent claudication or severe ischemia threatening the leg. Guidelines recommend bypass surgery for high-risk patients with long (>25 cm) lesions of the superficial femoral artery (SFA), when an autologous vein is available and life expectancy is >2 years (1). Over the years, endovascular therapy (EVT) has also become an established treatment method. Initially, EVT was feasible only for short and non-complex lesions; however, the development of dedicated tools—such as directional atherectomy devices, lasers, reentry devices, interwoven nitinol stents, and others—has enabled treatment of long and calcified infrainguinal lesions with variable results. Notably, calcified lesions are prevalent in the SFA, and lack a defined strategy (2, 3). Other risk factors for early failure, such as flow limiting dissection and elastic recoil, usually require the use of a nitinol self-expanding stent in the femoropopliteal segment. However, this strategy cannot be applied in the below-the-knee (BTK) vasculature due to unsatisfactory results, although a meta-analysis of randomized trials indicated that use of a drug-eluting stent seems to significantly improve mid-term results in terms of patency and amputation-free survival (4). Therefore, the EVT of SFA and BTK lesions should include optimal vessel preparation to limit dissection, gain lumen, and avoid elastic recoil. The use of a drug-coated balloon (DCB), drug-eluting stent (DES), or interwoven nitinol stent requires proper dilation using a long inflation and specific devices (5).

In this narrative review, we aimed to describe the current options for preparing a vessel in the SFA and BTK segment for drug-eluting technologies and for a specific self-expanding stent in the popliteal artery.

## Vessel Preparation and Device Selection

Vessel preparation is the first step after crossing the lesion with a guidewire. Depending on the lesion type (e.g., stenosis or occlusion), subintimal or endoluminal recanalization may be performed, and this choice affects the options for vessel preparation. Indeed, the subintimal space contraindicates a specific atherectomy device and limits the effects of specialized balloons. Therefore, it is advantageous to use the endoluminal approach when possible, to have full options. Notably, the armamentarium is growing, increasing both the indications for EVT and the technical success rate. However, these devices are usually very expensive and physicians should be aware of their costs before their use.

Two characteristics must be considered before selecting the optimal device for vessel preparation. The presence of heavily eccentric calcification requires vessel lumen debulking using atherectomy devices. On the other hand, long lesions with limited calcification and small diameter are more prone to dissection. Specialty balloons, e.g., scoring or encased balloons, may limit the rates of dissection and bail-out stenting. Device selection is also driven by the interventionalist's preference. Before the procedure, lesion evaluation by CT angiogram is essential to grade the calcification, although there is currently no well-accepted scale. Device selection can be anticipated before the procedure, to avoid use of a non-dedicated tool.

## Prevention of Dissection and Recoil

**Figure 1:**
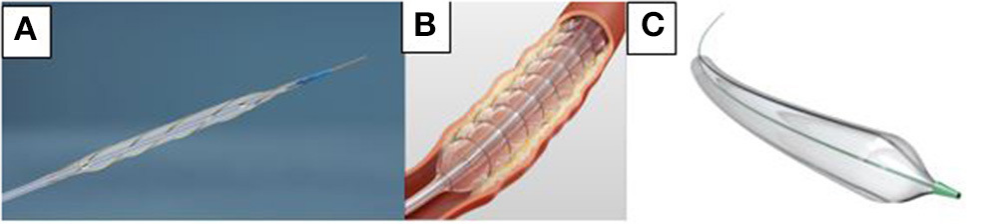
Specialized PTA Balloons. **(A)** Angiosculpt (BD Bard, New Jersey, USA). **(B)** Chocolate PTA Balloon (Medtronic, Santa Rosa, CA, USA). **(C)** Ultrascope (BD Bard, New Jersey, USA).

Using a specialty balloon can help limit the dissection and bail-out stenting rates, and can increase the drug uptake into the vessel wall after DCB dilation. Specialty balloons comprise three categories: cutting balloons, scoring balloons, and minimal trauma balloons (Figure 1). Cutting balloons include very sharp metal blades at their surface, which is useful for cutting the atherosclerotic plaque at a specific location. Scoring balloons have wires or polymer running over them, which significantly increase the pressure at specific points associated with precise rupture of the plaque. Finally, minimal trauma balloons correspond to the Chocolate PTA Balloon (Medtronic, Santa Rosa, CA, USA). This semi-compliant balloon is encased in a nitinol cage, creating a series of grooves and pillows that limit the propagation of dissection into the vessel wall. During inflation, the cage enables the balloon to dilate in a controlled manner, thus reducing overinflation and torsion, which promote dissection.

### Cutting Balloons

Cutting balloons were first used in coronary stenosis, but their indication has been extended to include infrainguinal steno-occlusive disease (6), resistant renal artery stenosis in pediatrics (7), and arteriovenous fistulae (8). Their use is limited in resistant fibrotic stenosis, and is mainly observed in vascular access for hemodialysis. A recent meta-analysis showed that 6-month target lesion patency was significantly higher in the percutaneous cutting balloon arm compared to with conventional and high-pressure balloon angioplasty (67.2 vs. 55.6%; 95% CI, 0.05–0.19; *P* < 0.05) (8).

### Scoring Balloons

Scoring balloons were developed by exploiting the concept of selected and precise rupture of the atheroma using microblades. The semi-compliant scoring balloons may be enveloped by multiple helical scoring elements (Angiosculpt™; Philips, San Diego, USA) or longitudinal wires (Ultrascore™; BD Interventional, Tempe, USA) and embedded with a rigid polymer (Advance Enforcer™; Cook Medical, Limerick, Ireland) or serrated strips (Serranator Alto™; CagentVascular, Wayne, USA). Limited evidence supports the efficacy of scoring balloons in femoropopliteal lesions. The lack of a randomized controlled trial (RCT) prohibits the conclusion that scoring balloons are more effective than standard balloon angioplasty. Nevertheless, some registries define the indications, safety, and efficacy of scoring balloon use in *de novo* lesions and in-stent restenosis.

The most cited registry is the single-center PANTHER Registry from Heidelberg, which examined the treatment of femoropopliteal lesions with the Angiosculpt™ PTA scoring balloon. It included 124 calcified *de novo* and restenotic lesions in 101 consecutive patients with claudication (65.3%) and critical limb ischemia (34.7%) (9). Three arms were investigated, according to the treatment selected at the interventionist's discretion. The first arm included lesions treated by scoring balloon alone (37.1%), the second scoring balloon followed by drug-eluting balloon (32.3%), and the third scoring balloon plus stent deployment (30.6%). The technical success of scoring balloon angioplasty was 100%. Overall primary patency (PP) at 12 months was 81.2% and did not significantly differ between the treatment groups. Nevertheless, it should be noted that the rate of occlusions were significantly higher in the combination group (17.5 vs. 2.2%). Obviously, PP was higher in patients with claudication (83.3%) vs. severe ischemia (73.7%). The 1-year overall amputation-free survival rate was 82.2%. Clinical success was evaluated by Rutherford class and ankle-brachial index (ABI), revealing significant improvement at 6 months and 1 year. The authors argued that lesion length was the major predictor of patency, with unfavorable results in long lesions (>10 cm). Notably, the recently released longer versions of scoring balloon might be more favorable for treating long SFA lesions.

The Serranator™ Alto PTA Serration balloon catheter is a recently developed semi-compliant scoring balloon with four embedded metal serrated strips, which was first used in femoropopliteal lesions. The interrupted scoring elements penetrate the intima, and possibly also the media, creating linear longitudinal interrupted serrations along the endoluminal surface. The PRELUDE study is a single-arm open-label prospective investigation of the technical feasibility and efficacy of using this scoring balloon in patients with peripheral arterial disease, Rutherford class 2–4 (10). This study included only 25 patients, precluding the observation of any difference in mean lumen gain between severely calcified and less calcified lesions (3.45 vs. 3.33 mm). Only one chronic total occlusion (CTO) required bail-out stenting due to grade D dissection. In all cases, optical coherence tomography (OCT) and intravascular ultrasound (IVUS) revealed serrations. This device is also under investigation in BTK lesions in the PRELUDE BTK study, for which recruitment is completed and results are awaited (ClinicalTrials.gov identifier NCT03693963).

The Advance Enforcer™ 35 focal-force PTA balloon catheter contains four polymer elements incorporated into its longitudinal axis. These plastic elements are incorporated during a single extrusion process, thus avoiding seams or joints, and providing focal force upon inflation to aid in lesion dilation. A single-center prospective feasibility study explored this scoring balloon's short-term performance to reduce stenosis of a mature native arteriovenous fistula (AVF) (11). Only one patient required an adjunctive treatment. Residual stenosis was <30% in 64.1% of patients, and <50% in 92.3%. These promising results must be confirmed in a RCT comparing this scoring balloon vs. angioplasty with regards to procedural outcomes and need for adjunctive therapy (ClinicalTrials.gov identifier NCT03552289).

The Vascutrak™ balloon (BD interventional, Tempe, USA) is a semi-compliant balloon with two external wires that deliver focused force along the length of the balloon. It was tested in a single-center study including 29 consecutive patients with symptomatic PAD due to femoropopliteal stenosis or occlusion (12). EVT comprised vessel preparation using a Vascutrak™ balloon, and subsequent DCB. At 2 years, no clinically driven target lesion revascularization (CD TLR) was observed, and only one patient had binary restenosis. The clinical success was significant, with over 90% of patients exhibiting improvement after 6 and 12 months, according to Rutherford classification. The next generation is represented by the Ultrascore™ Focus Force PTA balloon, which includes one wire embedded on the balloon surface and another working wire placed 180 degrees apart, designed to fracture the plaque at lower inflation pressures. A real-world multicenter registry is currently in progress to evaluate the clinical use of this scoring balloon for treatment of stenotic lesions of the femoropopliteal and infra-popliteal segments (ClinicalTrials.gov identifier NCT03193619).

### Minimal Trauma Balloon Catheter

Barotrauma is a major risk factor for dissection or perforation. Minimal trauma balloon catheters, such as the Chocolate PTA Balloon, are useful for preventing flow-limiting dissection and bail-out stenting. This balloon is not a scoring balloon, as the wires running at the surface do not come in contact with the vessel wall. Three major studies have reported results using different strategies. The Chocolate BAR registry included 262 patients treated with this balloon, without adjunctive DCB (13). The mean lesion length was 8.3 ± 5.9 cm, and acute luminal grain was 2.8 ± 0.7 mm. Severe or moderate calcification was observed in 63.4% of all lesions. Bail-out stenting was performed for residual stenosis of >30% in 1.6% of patients. Angiographic Core Laboratory revealed no flow-limiting dissection grades E or F.

A single-center prospective single-arm study evaluated the use of Chocolate PTA balloon, systematically followed by DCB, in 84 patients with femoropopliteal lesions (14). The mean stenosis length was 69.4 ± 30 mm, and CTO was present in 65.5% of the limbs, with a mean length of 186 ± 82 mm. The bail-out stenting rate was 9.5%, using only drug-eluting stents (DES). Freedom from TLR was 97.6% at a mean follow-up of 12.3 months. The authors mention that arterial wall dilatation prior to DCB use is very important, and should be performed using minimal barotrauma balloons (e.g., Chocolate) to limit the bail-out stenting rate.

Finally, the drug-coated chocolate balloon (Chocolate Touch™; QTVascular, Singapore) received CE mark approval in July 2015. Its safety and effectiveness were evaluated in the ENDURE trial, which included 67 patients with PAD Rutherford class 3–5 (15). The mean lesion length was 73 mm, and 54.3% of patients showed moderate-to-severe calcification. CTO was observed in 33.3% of patients. The bail-out stenting rate was only 1.4% after dilatation using Chocolate Touch™, and no flow-limiting dissection occurred. The 12-month PP was 89.9%. This balloon is currently being evaluated in the first FDA Investigational Device Exemption (IDE) pivotal RCT against a competing product (Lutonix; BD Interventional, Tempe, USA). Final data collection is expected in December 2020 (ClinicalTrials.gov identifier NCT02924857).

## Percutaneous Atherectomy

**Figure 2:**
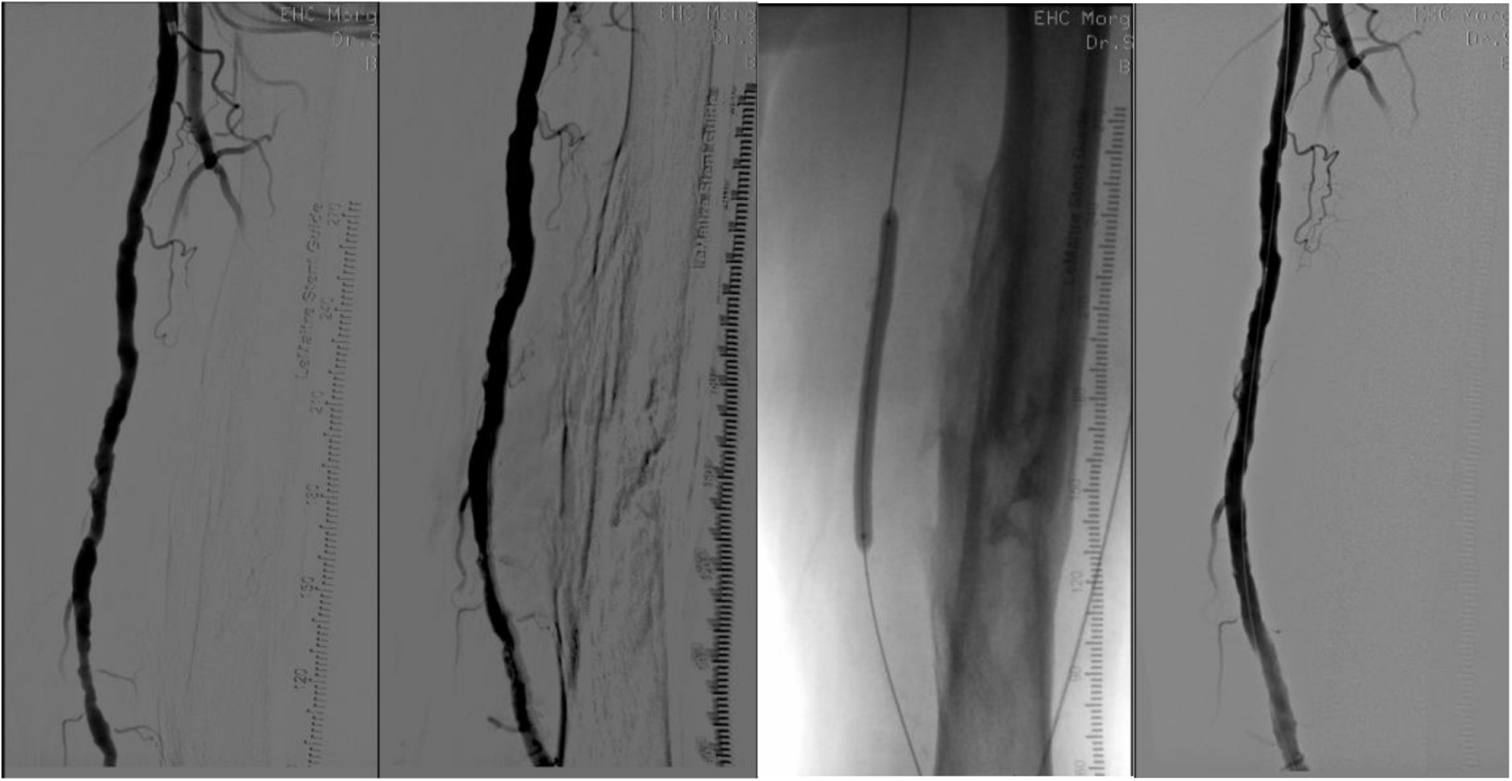
From left to right. (1) Long superficial femoral artery lesion; (2) After directional atherectomy, filter is in the popliteal artery (not seen); (3) DEB angioplasty; (4) Final result.

When lumen debulking is required due to coraliform calcification, percutaneous atherectomy is an option to gain lumen and limit dissection and provisional stenting. Depending on the selected device, atherectomy may be directional (SilverHawk™, TurboHawk™, Hawkone™, Pantheris™), hybrid (Phoenix™), rotational (Jetstream™), or orbital (Diamondback360® Peripheral Orbital Atherectomy), or may use laser technology for ablation (Turbo-Elite™ laser atherectomy catheter). Details are listed in Table 1. A case of directional atherectomy is shown in Figure 2. The use of a filter is required only in directional atherectomy to avoid distal embolization, which increases the cost and time of the procedure.

**Table 1:** Atherectomy devices.

**Device (company)** **names**	**Mechanism**	**Sheath** **compatibility**	**Clinical trial** **(study design)**	**Number** **of patients**	**Lesion characteristics** **(mean ± SD)**	**Outcomes**
**DIRECTIONAL ATHERECTOMY**						
SilverHawk (Medtronic, Minneapolis, MN, USA)	Single directional cutter with nosecone	6–8 Fr	DEFINITIVE LE (16) (prospective, single -Arm, international)	800	LL: 8.3 ± 5.5 cm CTO: 21% Calcification: 40%	Bail-out stenting rate: 3.2% Perforation: 5.3% Distal embolization: 3.8% Primary patency at 1 year : 78% Functional clinical outcomes at 12 months: Improvement in all categories (p < 0.05)
TurboHawk (Medtronic, Minneapolis, MN, USA)	4 contoured blades with nosecone	6–8 Fr	DEFINITIVE Ca^++^ (17) (prospective, single -arm, multicenter)	133	LL: 39 ± 27 CTO: 18% Calcification: 81%	Bail-out stenting rate: 4.1% Distal embolization: 1.7% <50% residual stenosis: 92%
HawkOne (Medtronic, Minneapolis, MN, USA)	4 contoured blades with nosecone	6–7 Fr	DEFINITIVE AR (prospective, multi -center RCT)	102 (*N* = 48 DA+DCB) (*N* = 52 DCB)	LL: 10.6 ± 4.4 cm Calcification: 20.5% (DA+ DCB) 18.5% (DCB)	Flow-limiting dissection: 2.1% (DA+DCB) 18.5% (DCB) Distal embolization: 8.0% (DA+DCB) 0% (DCB) Primary patency 82.4% (DA+DCB) 71.8% (DCB) Improvement of at leat 1 RCC At one year: 85.4% (DA+DCB) 93.9% (DCB)
Pantheris OCT (Avinger Inc., Redwood City, CA, USA)	Rotating cutter blade with internal OCT fiber	7–8 Fr	VISION (18) (prospective, single -arm, multicenter)	158	LL: 5.3 ± 4 cm CTO: 20.2% Calcification: 78.3%	Bail-out stenting rate: 5.1% Distal embolization: 2%
**HYBRID ATHERECTOMY**						
Phoenix atherectomy system (Philips, Brussels, Belgium)	Front cutter with Archimedes screw	5–7 Fr	EASE (19) (prospective, single -arm, multicenter)	128	LL: 3.4 ± 2.9 cm	Adjunctive therapy: 85% Freedom from TLR at 6 months: 88%
**ROTATIONAL ATHERECTOMY**						
Jetstream (Boston Scientific, MA, USA)	5 rotational front-cutting blades	7 Fr	Pathway PVD (20) (prospective, single -arm, multicenter)	172	LL: 2.7± 2.4 CTO: 31% Calcification: 51%	CD TLR at 1 year: 26% Restenosis rate at 12 months: 38.2%
**LASER ATHERECTOMY**						
Turbo-Elite laser atherectomy (Philips, Brussels, Belgium)	Excimer laser	4–8 Fr	CELLO (21) (prospective, single -arm, multicenter)	65	LL: 5.6 ± 4.7cm CTO: 20% Calcification: 62%	Bail-out stenting rate: 23% PP at 12 months: 54% Freedom from TLR at 12 months: 76.9%
**ORBITAL ATHERECTOMY**						
Diamondback 360 (CSI, St. Paul, MN, USA)	Diamond-coated crown	4–6 Fr	COMPLIANCE 360 (22) (prospective, multi -center RCT)	50 *N* = 25 (POBA+OAS) *N* = 25 (POBA)	LL: 5.6 cm (POBA) 8.7 cm (POBA+OAS) Moderate to severe calcification: 44.4% (POBA+OAS) 45.5% (OAS) CTO: 18.5% (POBA+OAS) 21% (OAS)	Bail-out stenting rate: POBA+OAS: 5.3% POBA: 77.8% Freedom from TLR at 12 months: POBA+OAS: 81.2% POBA: 78.3% Rutherford classification: Baseline vs. 12 months: POBA+ OAS: 2.80 vs. 1.12 POBA: 2.92 vs. 0.55

*LL, lesion length; CTO, chronic total occlusion; RCT, randomized controlled trial; DA, directional atherectomy; DCB, drug-coated balloon; OCT, optical coherence tomography; CD TLR, clinically driven target lesion revascularization; PP, primary patency; POBA, plain old balloon angioplasty; OAS, orbital atherectomy system; RCC, Rutherford clinical category*.

## Intravascular Lithotripsy

The intravascular lithotripsy catheter (IVL; Shockwave Medical, Santa Clara, USA) comprises a dedicated balloon catheter associated with miniaturized and arrayed lithotripsy emitters to create a localized field effect that travels through the vessel wall. The waves selectively crack intimal and medial calcium. After a few cycles, the balloon is used to dilate the lesion at low pressure to gain lumen. A first prospective single-arm multicenter study (Disrupt PAD II study) was conducted to evaluate the IVL safety and effectiveness in femoropopliteal lesions in 60 subjects with PAD Rutherford classification 2–3 (23). The average lesion length was 76.9 mm, with moderate (8.3%) or severe (85%) calcification according to Peripheral Academic Research Consortium (PARC) definitions. The final residual stenosis was 24.2%, with an average acute gain of 3.0 mm. The bail-out stenting rate was 1.7%, with 8.4% flow-limiting dissection. No distal embolization occurred. At 12 months, the primary performance endpoint was 69.8%, the primary patency rate was 54.5%, and freedom from CD TLR was 79.3%. The major adverse event rate was 1.7%. To improve the level of evidence, a RCT is currently enrolling patients to evaluate the use of IVL+ DCB vs. DCB alone in calcified lesions (Disrupt PAD III study; ClinicalTrials identifier NCT02923193).

## Discussion

Nowadays, plain balloon angiography is rarely used to treat infrainguinal lesions, since stenting and drug-eluting technologies have significantly improved the mid-term and long-term results. Nevertheless, elastic recoil, flow-limiting dissection, calcification, and long lesions are still risk factors for early failure. Therefore, vessel preparation is mandatory to gain lumen and debulk calcification, and thus promote better stent deployment and drug deposition into the vessel wall. Many devices are available, but not all are reimbursed in every country. Interventionists should select the optimal device according to the preoperative evaluation based on imaging. In particular, calcification evaluation is crucial for deciding on a strategy. Scoring balloons are less effective in heavily calcified lesions, which require atherectomy for lumen debulking. The interventionist should select one or two scoring balloons and atherectomy devices. Experience is necessary to define indications, and to properly use these expensive devices. The current literature lacks the high-level evidence required, and additional studies are needed to compare all these devices and to optimize the indications for drug-eluting technologies or stenting. Scoring balloons and atherectomy should definitely not be used alone, as the results do not differ from with simple balloon angiography.

## Author Contributions

FS: manuscript redaction and literature research. HP: literature research and revision. RT: revision and edition. All authors: contributed to the article and approved the submitted version.

## Conflict of Interest

The authors declare that the research was conducted in the absence of any commercial or financial relationships that could be construed as a potential conflict of interest.
